# Supplementary material for: Contribution of cytotoxic CD8 T cells, neutrophils and type 1 interferon signaling to hyperinflammation in HIV-associated TB meningitis
Source: bioRxiv. 2026 Mar 23:2026.03.10.710544. Preprint. [Version 2] doi: 10.64898/2026.03.10.710544 (PMC13061046; doi:10.64898/2026.03.10.710544)
Supplement: Supplementary file 1 [file media-1.pdf]

Supplementary figures:

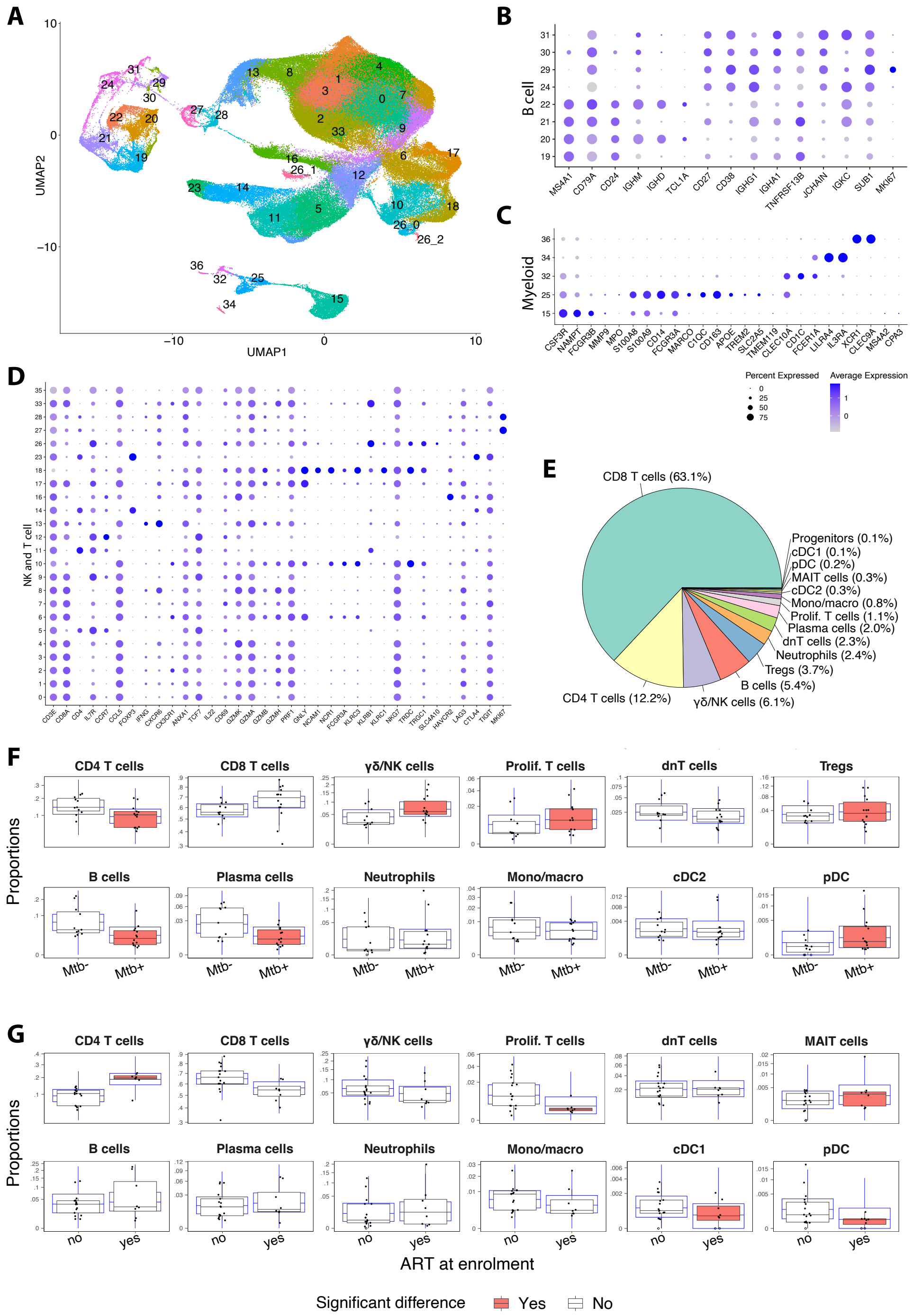

Fig. S1. All cells. (A) Unannotated UMAP of the 188,983 cells after predicted doublet and contamination removal at resolution 0.9. **(B)** Dot plot of original clusters classed as B or plasma cells based on canonical markers. **(C)** Dot plot of original clusters classed as myeloid cells based on canonical markers. **(D)** Dot plot of original clusters classed

as NK or T cells based on canonical markers. **(E)** Pie chart of proportions of coarse cell types after manual and automated annotation. **(F)** Comparison of cell composition by microbiological-confirmation using the sccomp R package adjusting for age and sex. The black boxplot is the observed data; the blue boxplot is simulated data using a posterior predictive distribution. **(G)** Comparison of cell composition by ART at enrolment using the sccomp R package adjusting for age and sex.

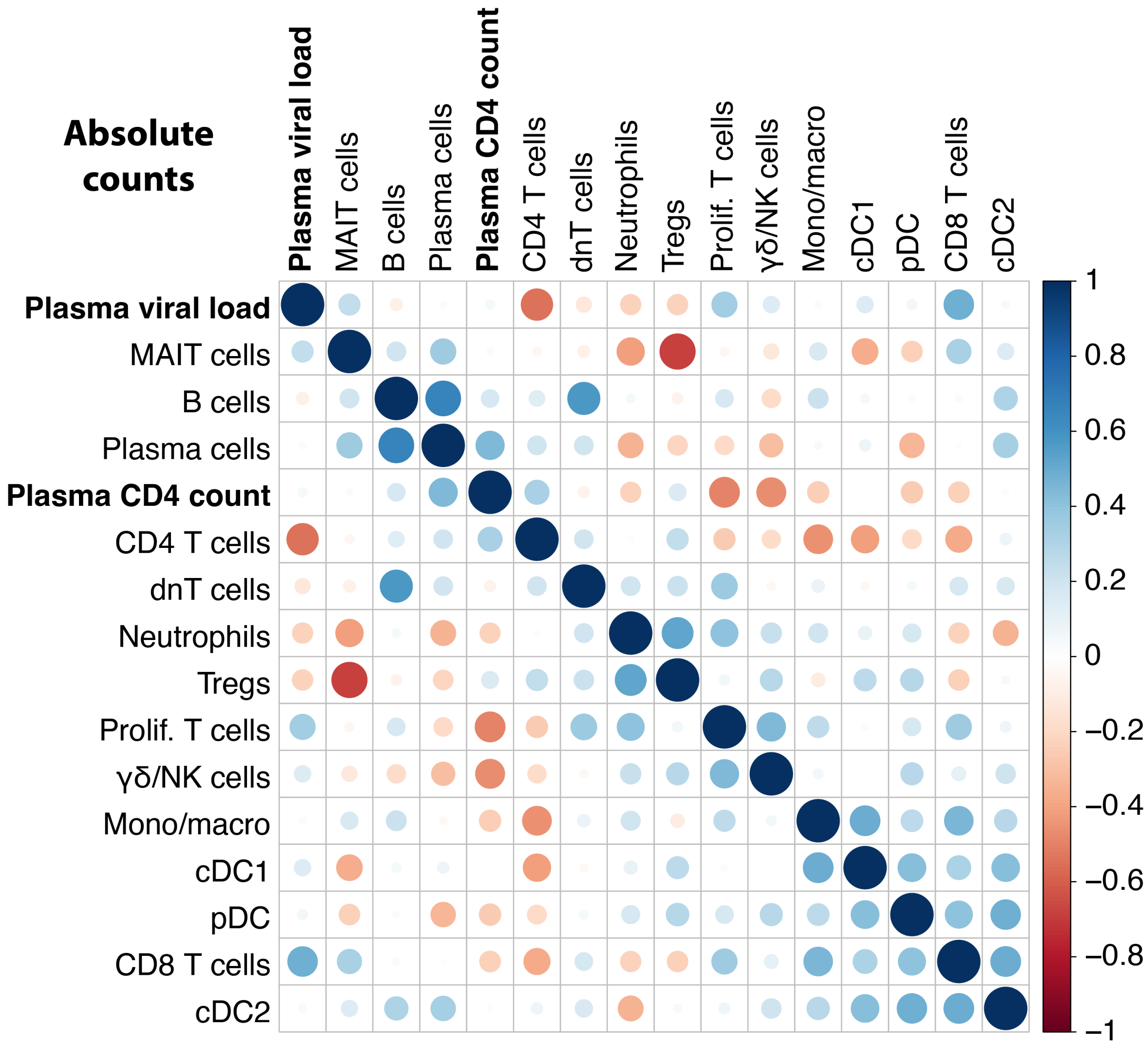

Fig. S2. Correlation matrix of cell types. Correlation matrix of absolute cell counts across the 25 patients calculated with Spearman's rank correlation. Laboratory plasma CD4 count (cells/mm^3) and HIV-1 viral load (copies/ml) highlighted in bold. Blue represents positive correlation and red negative correlation. Cell types ordered by hierarchical clustering.

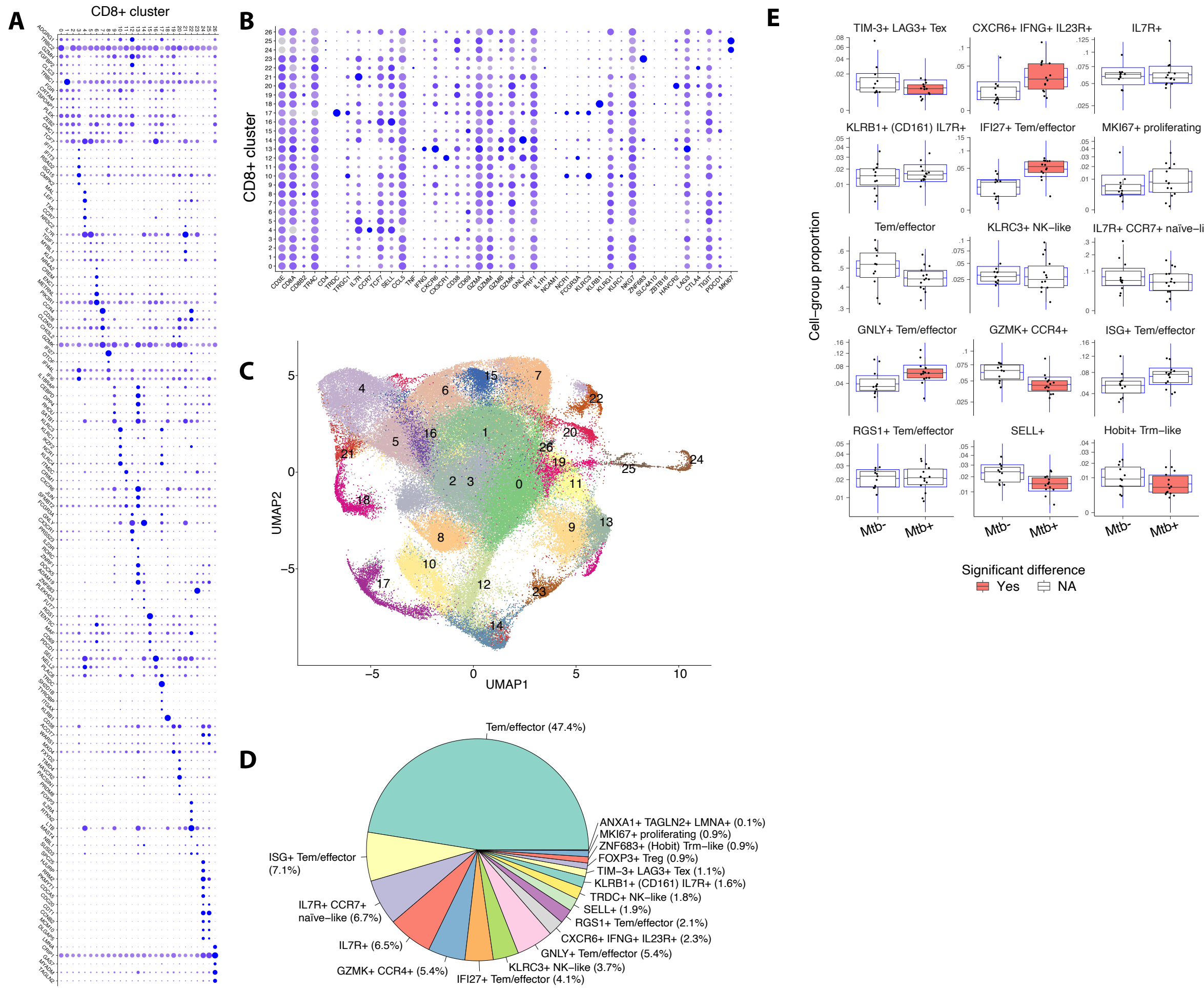

Fig. S3. CD8 T cells. **(A)** Dot plot of top 5 DEG per cluster. **(B)** Dot plot of canonical genes used to guide manual annotation. **(C)** Unannotated clustering of CD8+ T cells. Raw counts from 123,557 CD8A+ CD3+ cells were re-normalised, merged, integrated and clustered at resolution 0.8 to create 27 clusters. **(D)** Pie chart of CD8 T cell cluster proportions. **(E)** Comparison of CD8 T cell subcluster composition by microbiological-confirmation using the sccomp R package adjusting for age and sex. The black boxplot is the observed data; the blue boxplot is simulated data using a posterior predictive distribution. GXPU, GeneXpert Ultra. *IFNG*, interferon gamma; *GNLY*, granulysin; Tem, effector memory T cell.

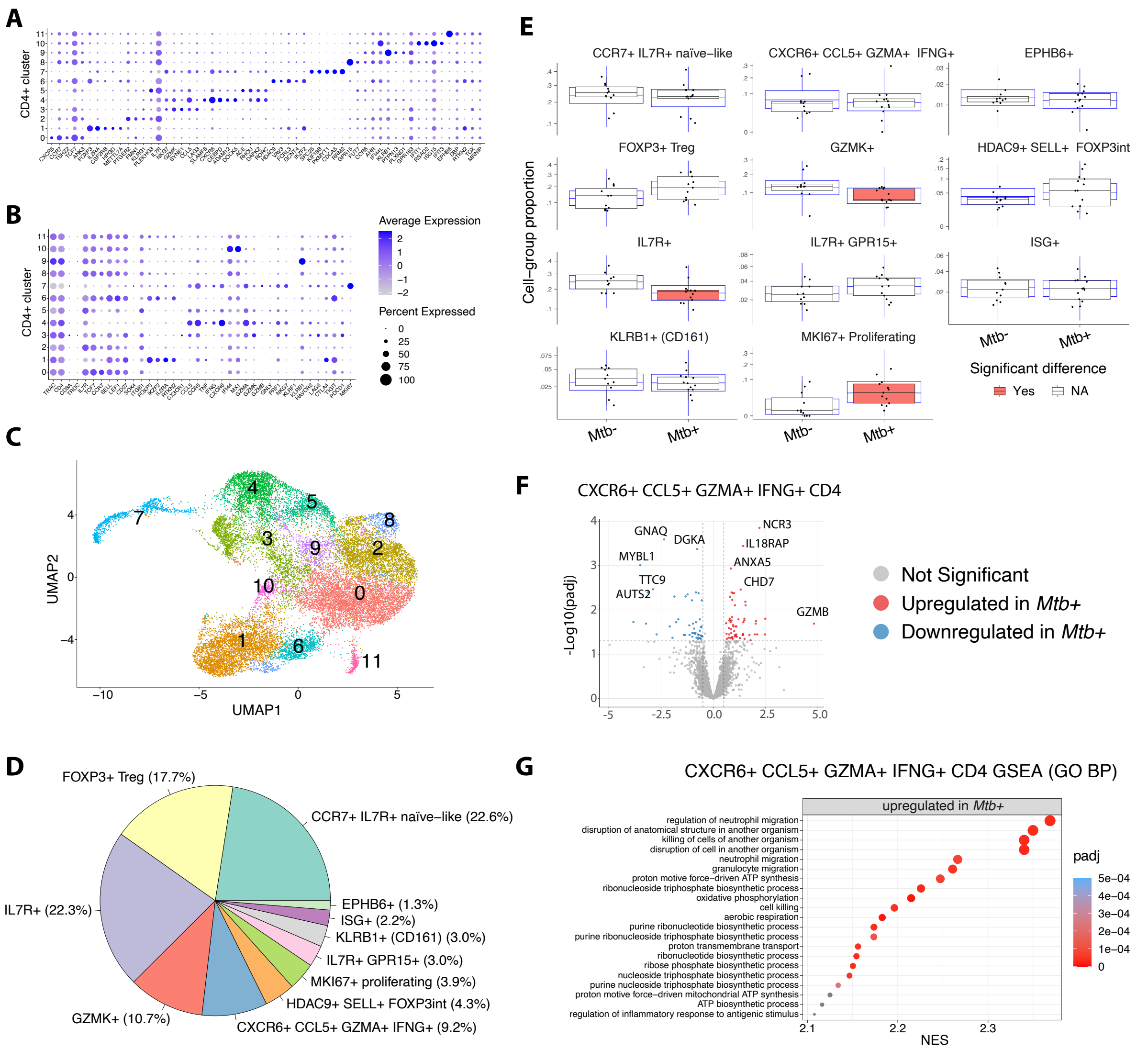

Fig. S4. CD4 T cells. **(A)** Dot plot of top 5 DEG per cluster. **(B)** Dot plot of canonical genes used to guide manual annotation. **(C)** Unannotated UMAP of CD4 T cells. Raw counts from 21,844 CD4+ CD3+ cells were re-normalised, merged, integrated and clustered at resolution 0.4 to create 12 clusters. **(D)** Pie chart of CD4 T cell cluster proportions. **(E)** Comparison of CD4 T cell subcluster composition by microbiological-confirmation using the sccomp R package adjusting for age and sex. The black boxplot is the observed data; the blue boxplot is simulated data using a posterior predictive distribution. **(F)** Volcano plot of DEG of CXCR6+ CCL5+ GZMA+ IFNG+ CD4 T cell by microbiological confirmation. **(G)** Gene set enrichment analysis of DEG by microbiological confirmation in the CXCR6+ CCL5+

GZMA+ IFNG+ CD4 T cell cluster done using the Gene Ontology Biological Processes gene set, ranked by log2 fold change. ISG, interferon-stimulated gene; *KLRB1*, killer cell lectin like receptor 1 (CD161); *EPHB6*, ephrin type-B receptor 6; *GZMA*, granzyme A; *IFNG*, interferon gamma.

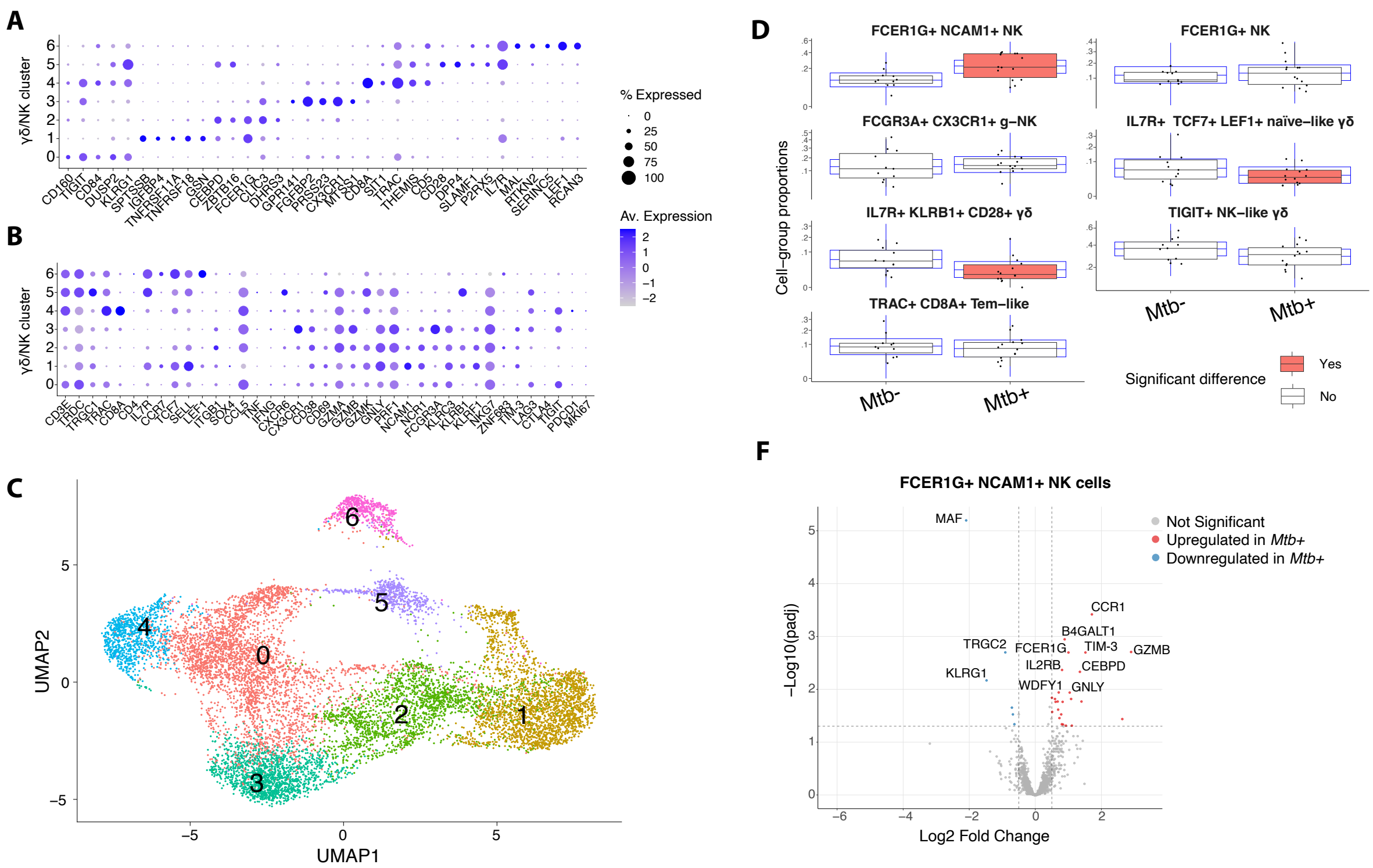

Fig. S5. NK and γδ cells. (A) Dot plot of top 5 DEG per cluster. **(B)** Dot plot of canonical genes used to guide manual annotation. **(C)** Unannotated UMAP of NK and γδ T cells. Raw counts from 11,483 NK and γδ cells were re-normalised, merged, integrated and clustered at resolution 0.2 to create seven clusters. **(D)** Comparison of NK and γδ T cell subcluster composition by microbiological-confirmation using the sccomp R package adjusting for age and sex. The black boxplot is the observed data; the blue boxplot is simulated data using a posterior predictive distribution. **(E)** Volcano plot of DEG for the FCER1G+ NCAM1+ NK cell cluster by microbiological confirmation. *FCER1G*, Fc fragment of IgE receptor, gamma chain; *NCAM1*, neural cell adhesion molecule 1.

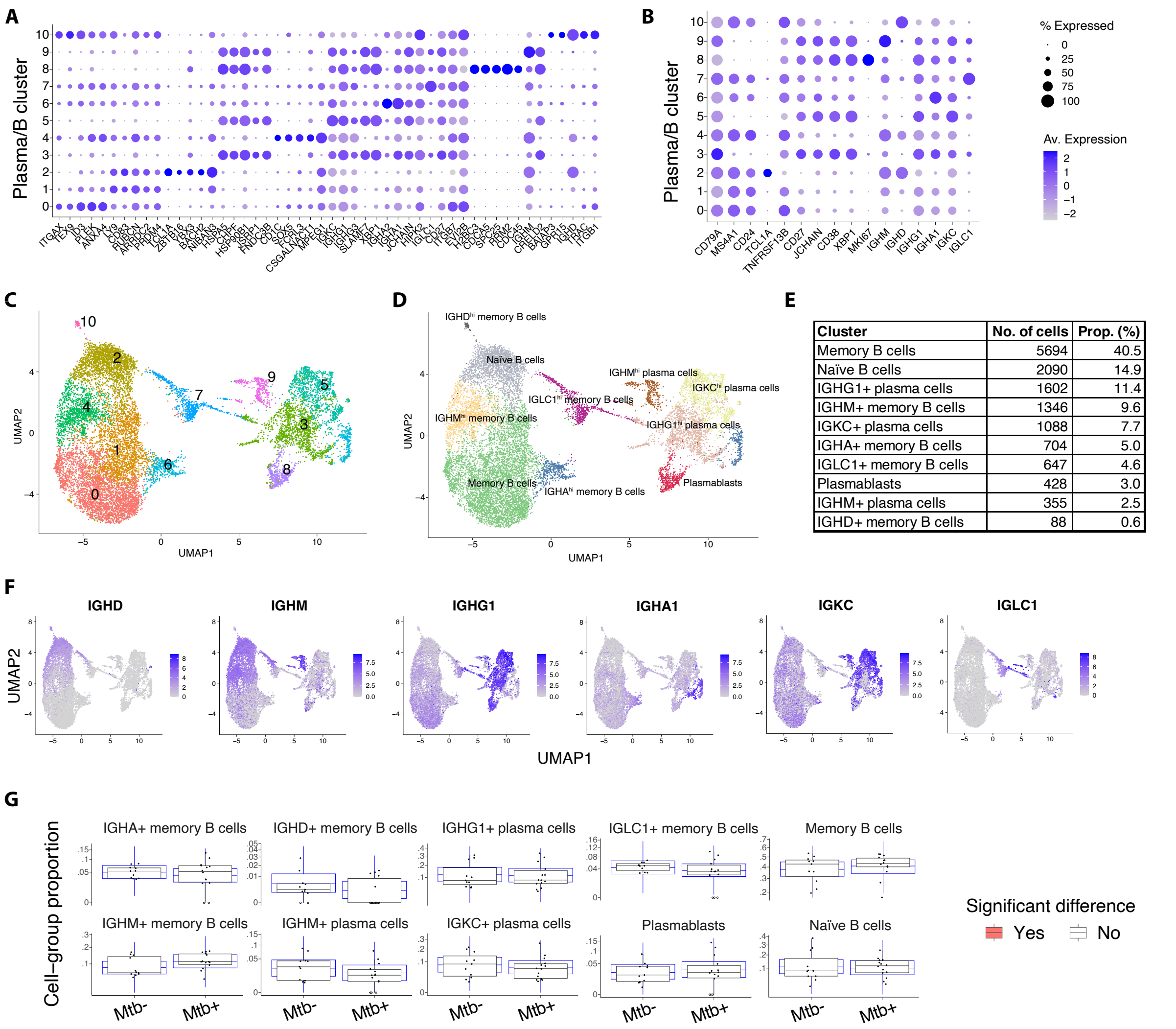

Fig. S6. B and plasma cell subclustering by microbiological confirmation. (A) Dot plot of top 5 DEG per cluster. **(B)** Dot plot of canonical genes used to guide manual annotation. **(C)** Unannotated UMAP of B and plasma cells. Raw counts from 14,042 B cells and plasma cells were re-normalised, merged, integrated and clustered at resolution 0.3 to create 11 clusters. **(D)** Annotated UMAP of 14,042 plasma and B cells. **(E)** Table of absolute numbers and proportions of B and plasma cell clusters. **(F)** Feature plots of immunoglobulin gene expression. **(G)** Comparison of B and plasma cell subcluster composition by microbiological-confirmation using the sccomp R package adjusting for age and sex. The black boxplot is the observed data; the blue boxplot is simulated data using a posterior predictive distribution. *IGHD*, IgD heavy

chain; *IGHM*, IgM heavy chain; *IGHG1*, IgG heavy chain 1; *IGHA1*, IgA heavy chain 1; *IGKC*, immunoglobulin kappa light chain constant; *IGLC1*, immunoglobin lambda light chain constant 1.

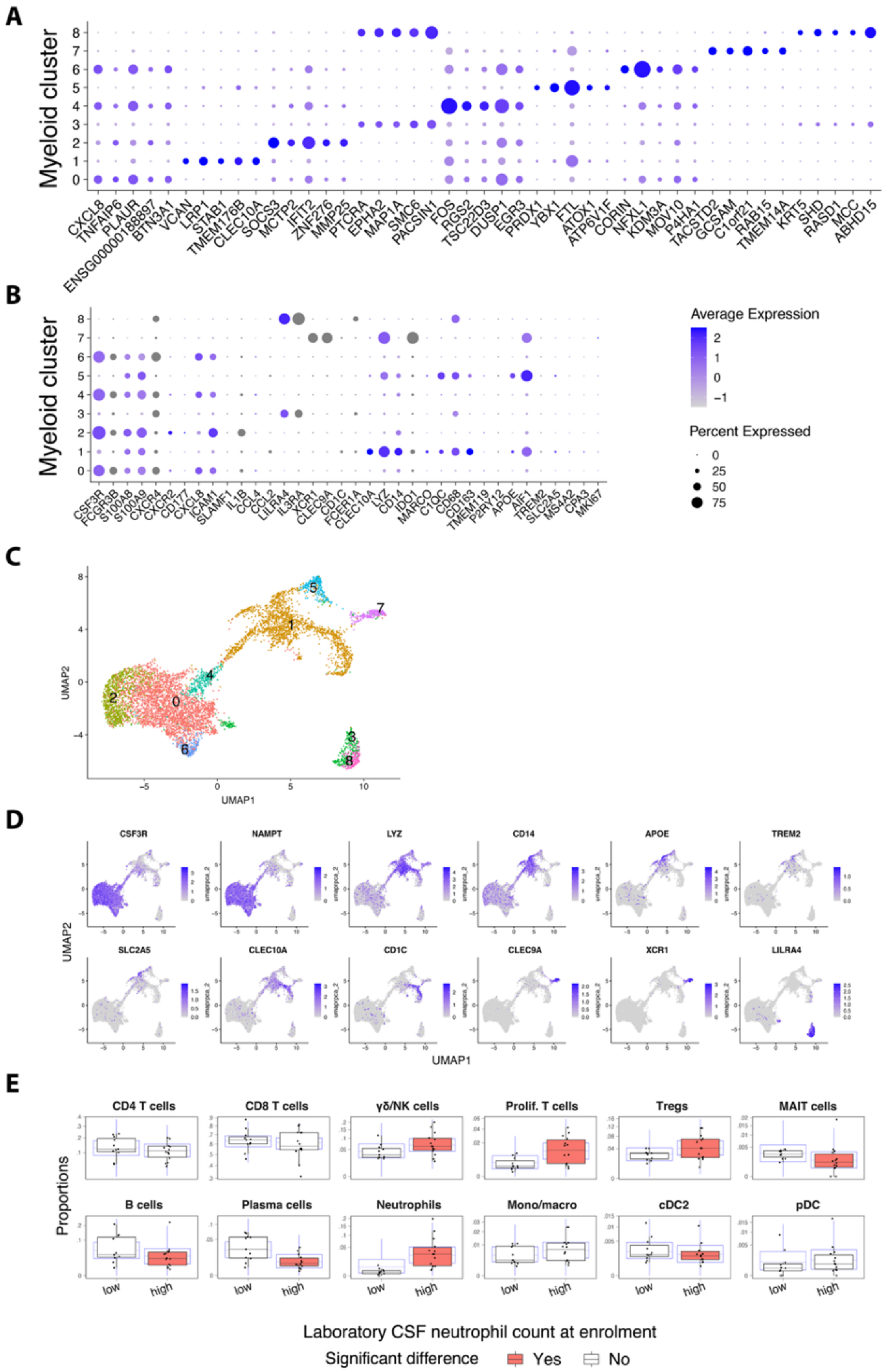
A
Myeloid cluster
CXCL8
TNFAIP6
PLAUR
ENSG00000188897
BTN3A1
VCAN
LRP1
STAB1
TMEM176B
CLEC10A
SOCS3
MCTP2
IFIT2
ZNF276
MMP25
PTCRA
EPHA2
MAP1A
SMC6
PACSIN1
FOS
RGS2
TSC22D3
DUSP1
EGR3
PRDX1
YBX1
FTL
ATOX1
ATP6V1F
CORIN
NFXL1
KDM3A
MOV10
P4HA1
TACSTD2
GCSAM
C1orf21
RAB15
TMEM14A
KRT5
SHD
RASD1
MCC
ABHD15
B
Myeloid cluster
CSF3R
FCGR3B
S100A8
S100A9
CXCR4
CXCR2
CD177
CXCL8
ICAM1
SLAMF1
IL1B
CCL4
CCL2
LILRA4
IL3RA
XCR1
CLEC9A
CD1C
FCER1A
CLEC10A
LYZ
CD14
IDO1
MARCO
C1QC
CD68
CD163
TMEM119
P2RY12
APOE
AIF1
TREM2
SLC2A5
MS4A2
CPA3
MKI67
Average Expression
Percent Expressed
C
UMAP2
UMAP1
D
CSF3R
NAMPT
LYZ
CD14
APOE
TREM2
SLC2A5
CLEC10A
CD1C
CLEC9A
XCR1
LILRA4
UMAP2
UMAP1
E
CD4 T cells
CD8 T cells
γδ/NK cells
Prolif. T cells
Tregs
MAIT cells
B cells
Plasma cells
Neutrophils
Mono/macro
cDC2
pDC
Proportions
low
high
Laboratory CSF neutrophil count at enrolment
Significant difference
Yes
No

Fig. S7. Myeloid cells. (A) Dot plot of top 5 DEG per cluster. **(B)** Dot plot of canonical genes used to guide manual annotation. **(C)** Unannotated UMAP of myeloid cells. Raw counts from 7,182 myeloid cells were re-normalised, merged, integrated and clustered at resolution 0.2 to create nine clusters. **(D)** Feature plots of myeloid cells with neutrophil canonical markers. **(E)** Comparison of coarse cell composition by laboratory CSF neutrophil count (high = ≥5 cells/mm^3, low = <5 cells/mm^3) using the sccomp R package adjusting for age and sex. The black boxplot is the observed data; the blue boxplot is simulated data using a posterior predictive distribution. *CSF3R*, colony-stimulating factor 3 receptor; *NAMPT*, nicotinamide phosphoribosyltransferase (visfatin); *LYZ*, lysozyme; *APOE*, apolipoprotein E; *TREM2*, triggering receptor expressed on myeloid cells 2; *SLC2A5*, solute carrier family 2 member 5 (GLUT5); *CLEC10A*, C-type lectin domain family 10 member A; *CLEC9A*, C-type lectin domain family 9 member A; *XCR1*, XC motif chemokine receptor 1; *LILRA4*, leukocyte immunoglobulin-like receptor subfamily A member 4.

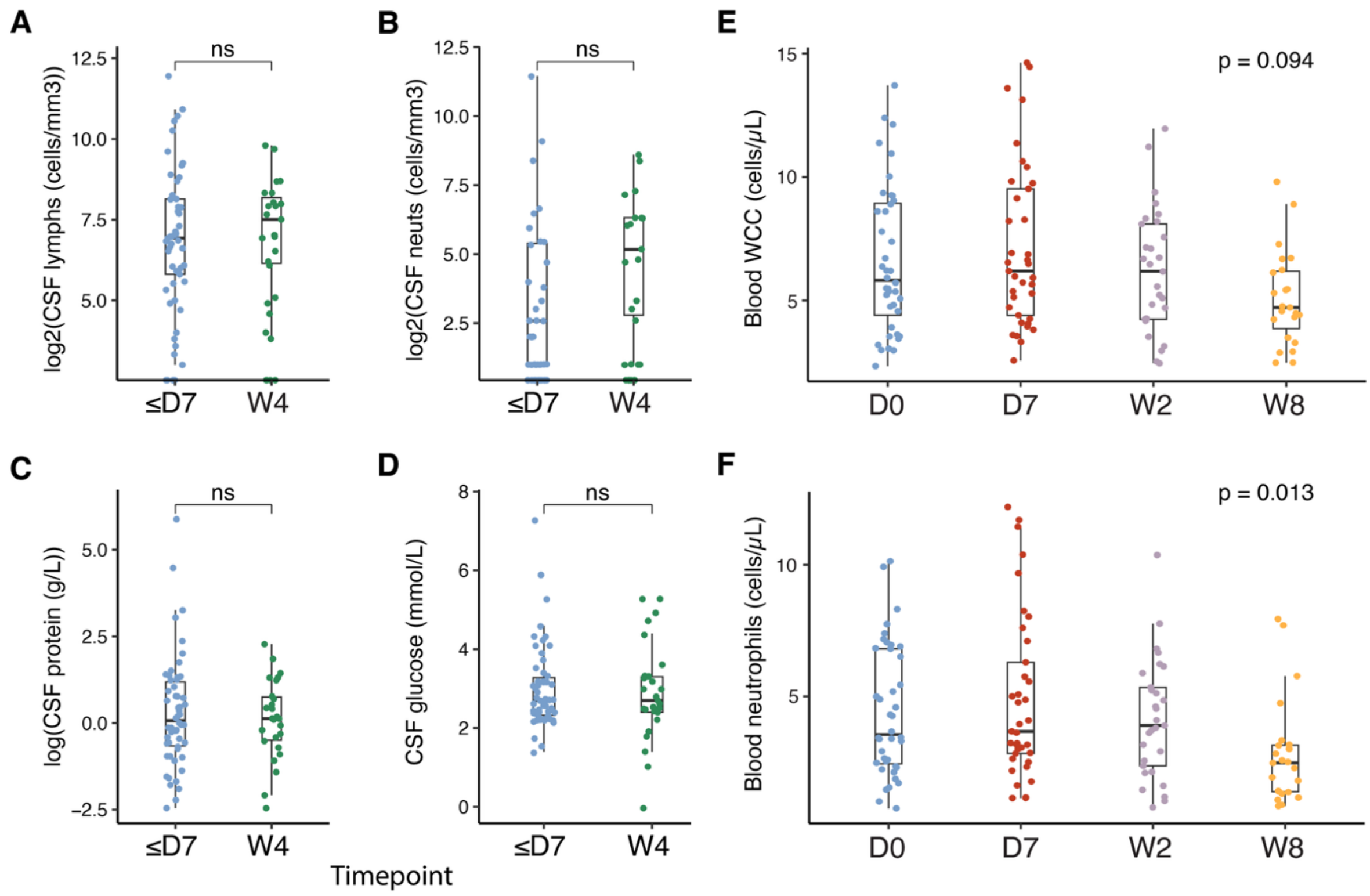

Fig. S8. Cell counts over time for participants in CSF and blood bulk RNA sequencing.

Comparison between early (≤7 days, n = 44) and late (week 4, n = 20) laboratory lymphocyte counts (A), neutrophil counts (B), protein (C) and glucose (D) for participants enrolled in INTENSE-TBM and LASER-TBM whose samples were used for CSF bulk RNA sequencing (patients = 53, samples = 64). +1 added to CSF cell counts, and +0.1 added to CSF protein to allow for log2 transformation of zero values. Comparisons between laboratory white cell count (E) and neutrophil count (F) at day 0 (n = 40), day 7 (n = 35), week 2 (n = 28) and week 8 (n = 22) for participants enrolled in LASER-TBM whose samples were used for blood bulk RNA sequencing (patients = 42, samples = 125). Comparisons made using Mann Whitney U to compare the two CSF timepoints and ANOVA to compare the four blood timepoints. ns = non-significant.

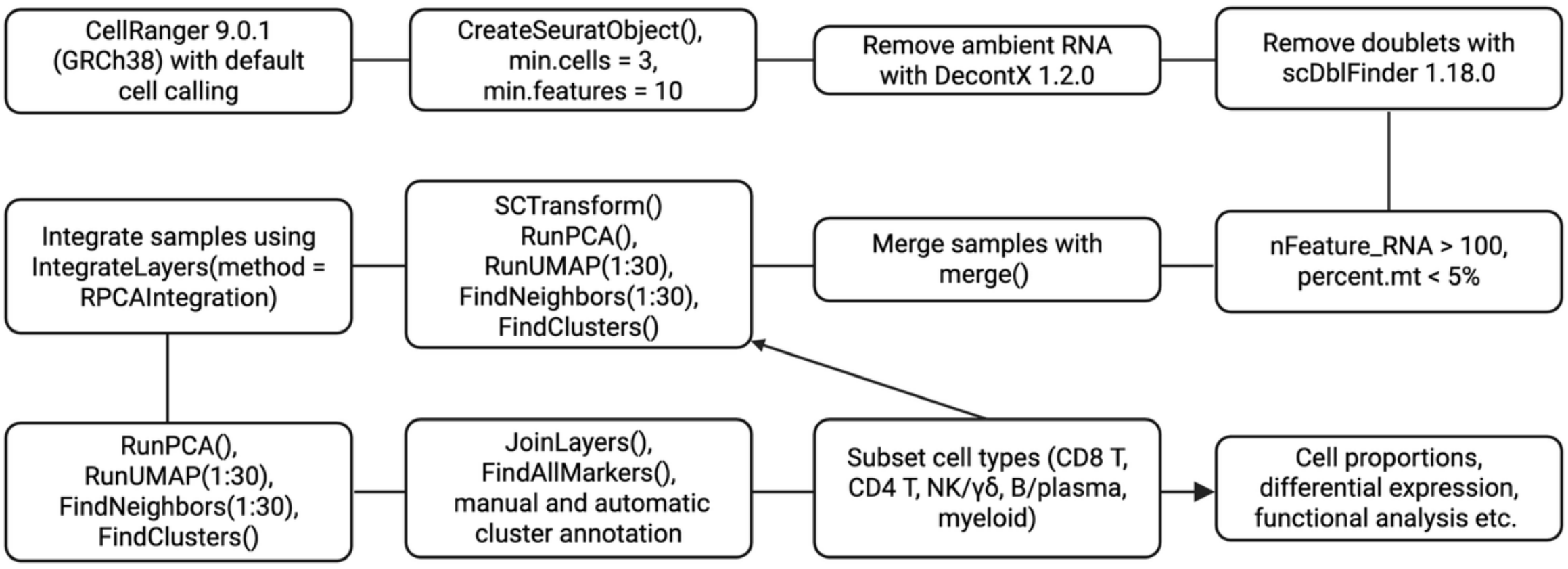

Fig. S9. 10x Flex single cell RNA sequencing bioinformatics workflow. Doublets refer to two or more cells that have been captured in the same droplet at the cell separation step, and therefore share the same cellular barcode. SCTransform refers to a normalisation and variance stabilisation technique using regularised negative binomial regression. RPCA, reciprocal principal component analysis.

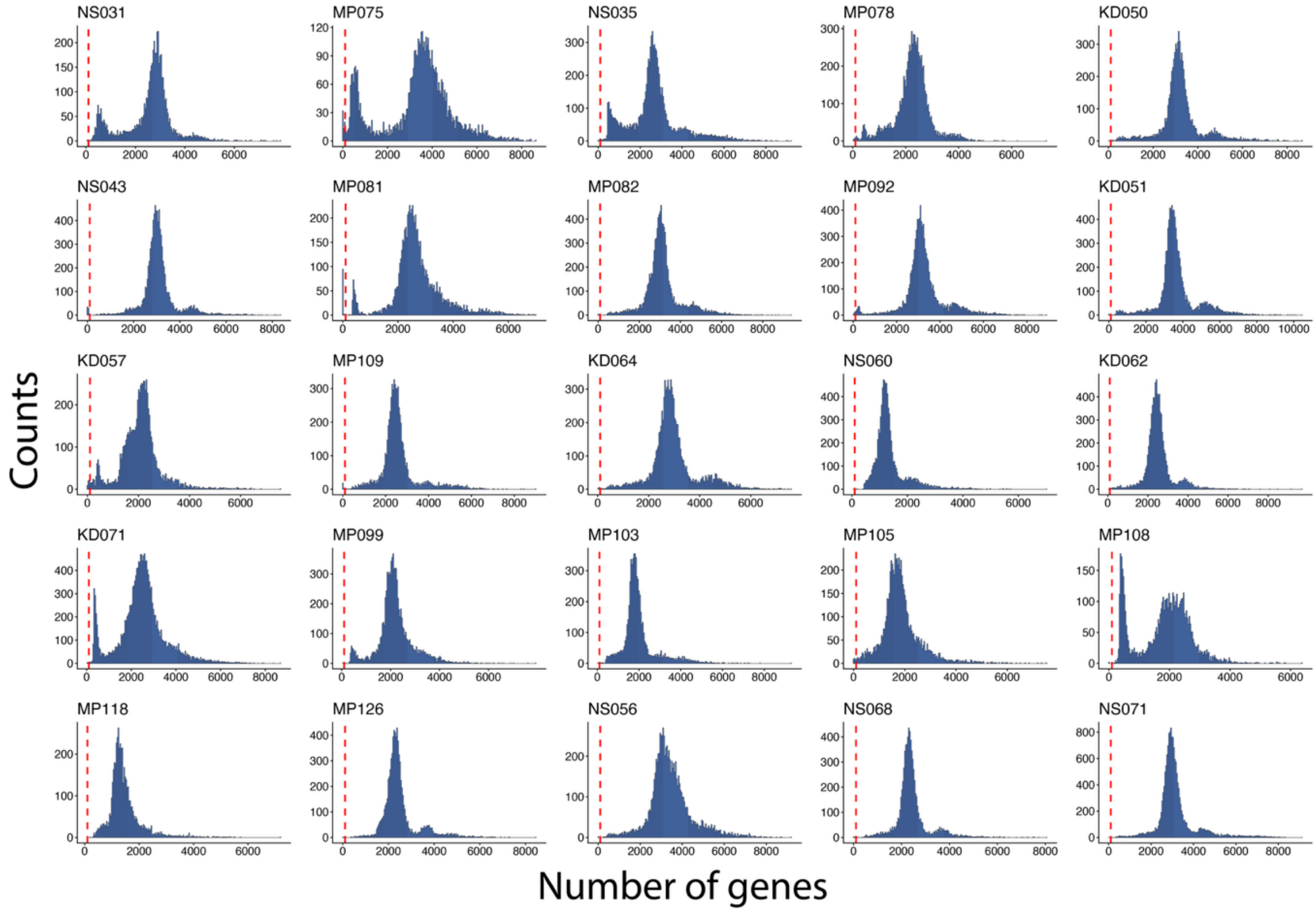

Fig. S10. Histograms of genes per cell. Histogram of each sample plotting the number of genes per cell (nFeature_RNA) vs. frequency. Red dotted line indicates the cut off of 100 genes/cell used for filtering. Each panel is one donor.

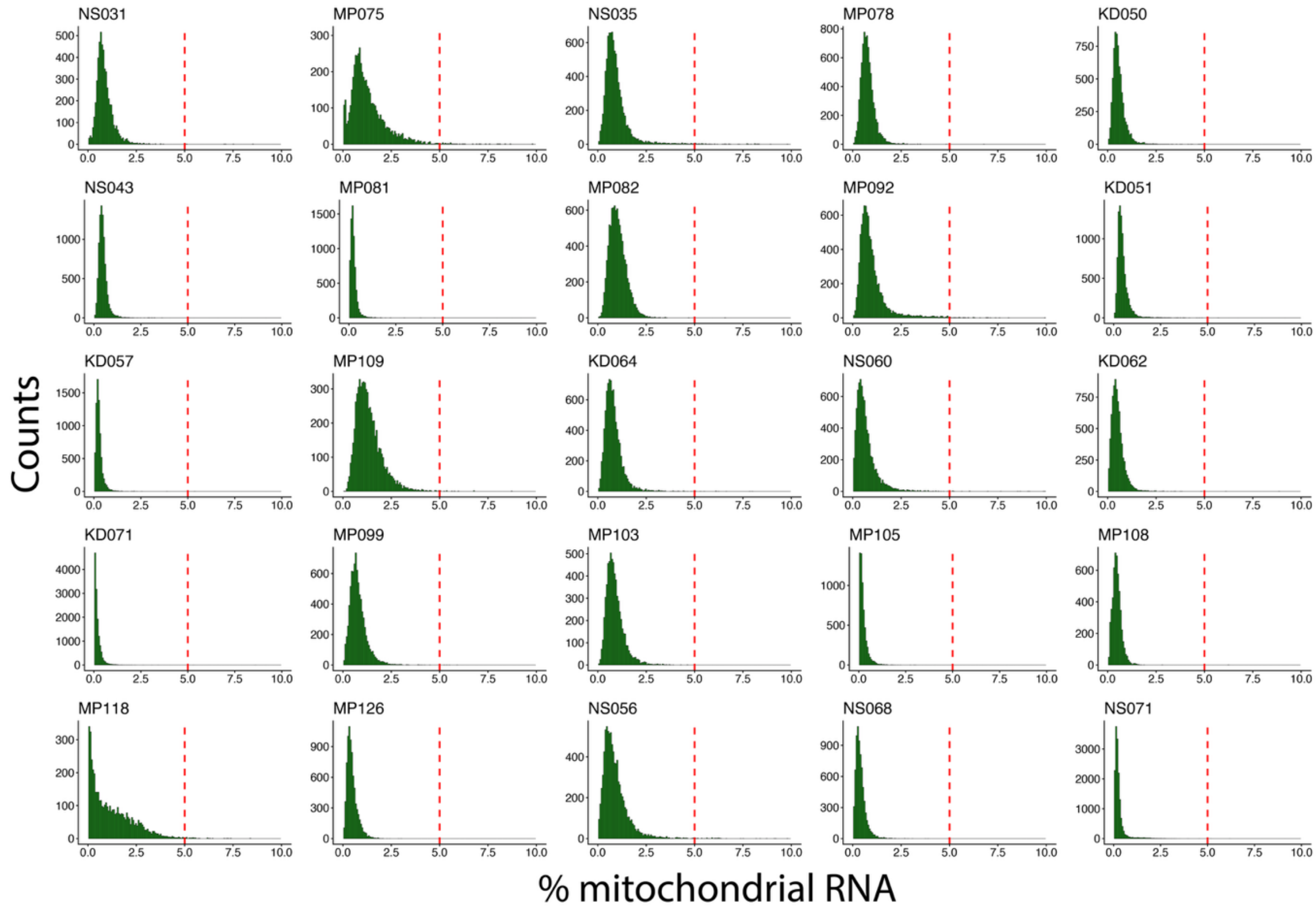

Fig. S11. Histograms of percentage of mitochondrial RNA per cell. Histogram of each sample showing the percentage of mitochondrial RNA per cell vs. frequency. Red dotted line indicates the cut off of 5% mitochondrial RNA used for filtering. Each panel is one donor.

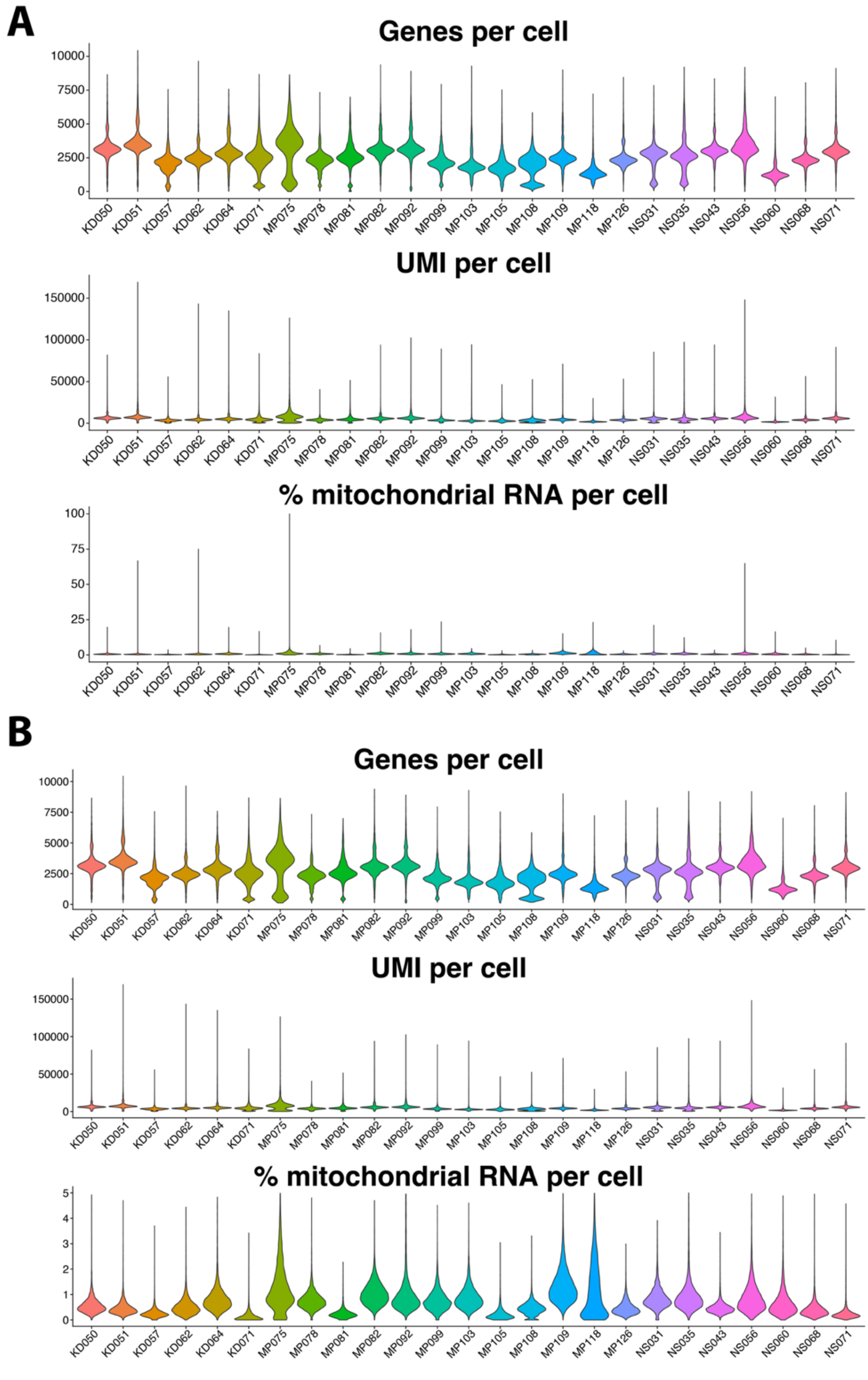

Fig. S12. Genes, UMI and percent mitochondrial genes per cell before and after filtering. A) Genes, UMI and percent mitochondrial genes per cell before filtering B) Genes, UMI and percent mitochondrial genes per cell after filtering cells with <100 genes and >5% mitochondrial RNA.

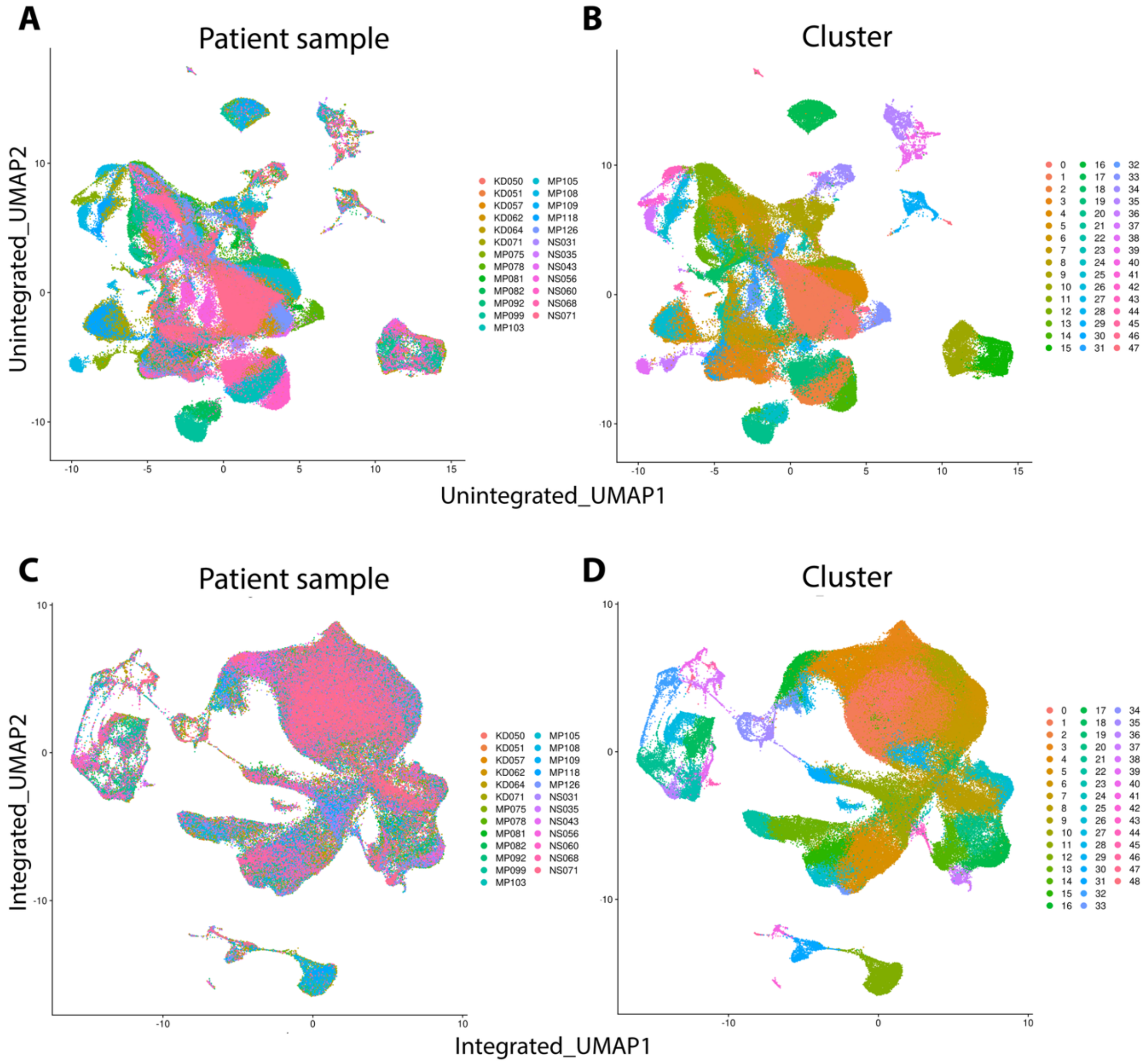

Fig. S13. Reciprocal PCA integration. (A) Unintegrated UMAP coloured by sample. **(B)** Unintegrated UMAP coloured by default clustering. **(C)** Reciprocal PCA integrated UMAP coloured by sample. **(D)** Reciprocal PCA integrated UMAP coloured by clustering at resolution 0.9. N.B. Sample colours are added by layer, with NS071 added last, meaning that later samples appear more prominent.

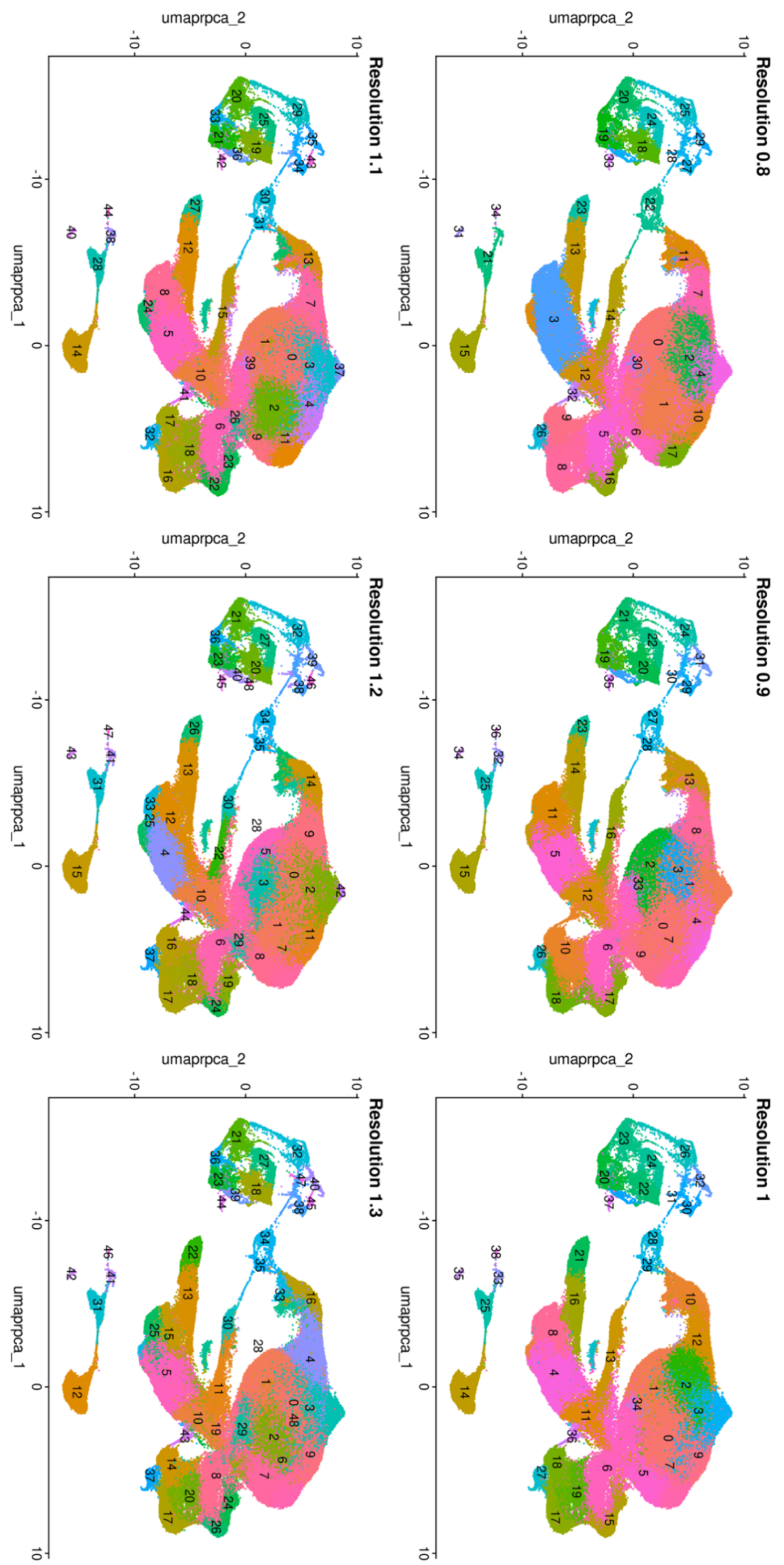

Fig. S14. Integrated UMAPs at selected cluster resolutions. UMAP at six resolutions using FindClusters(). Resolution 0.9 was used for annotation.

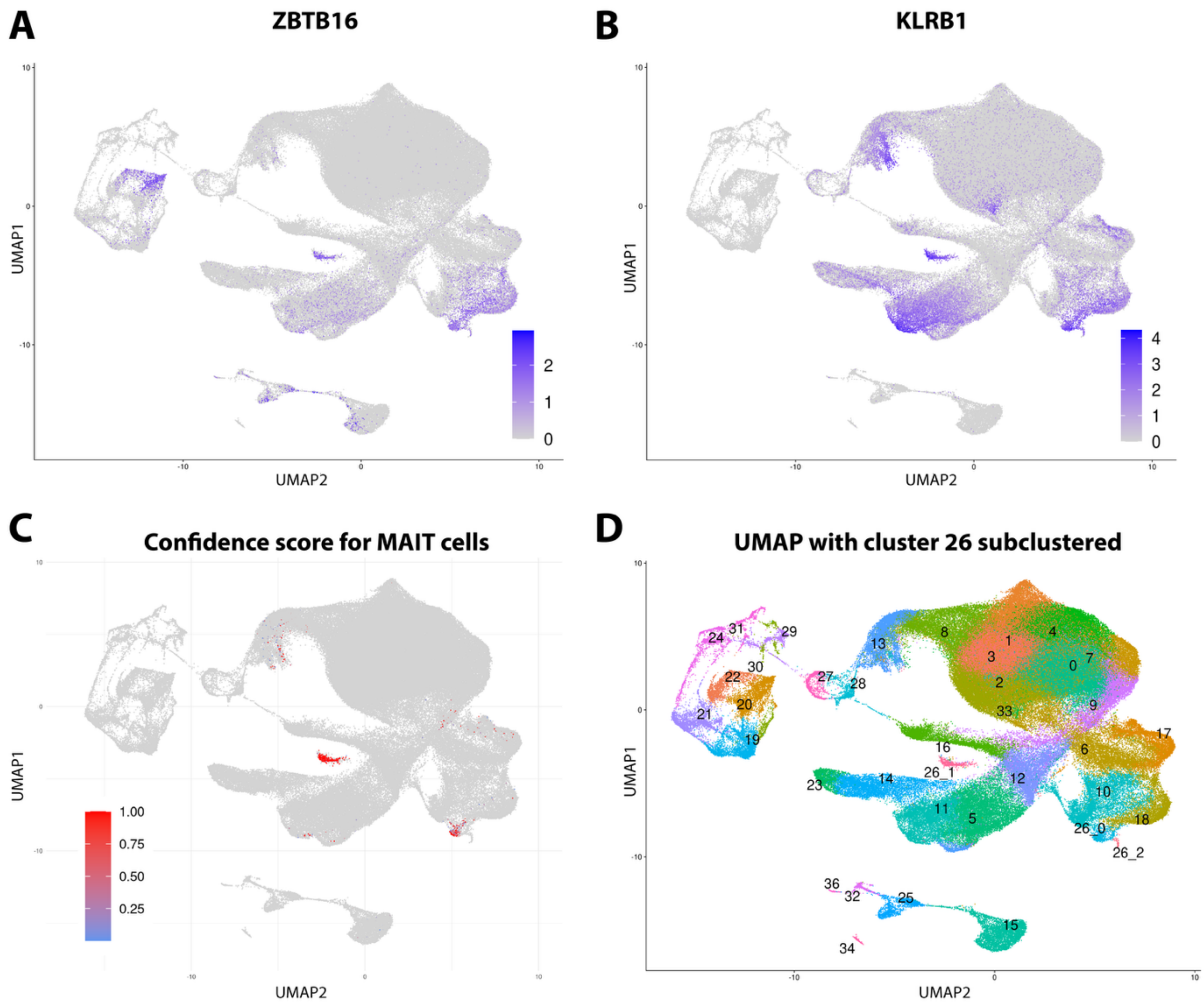

Fig. S15. Annotation of MAIT cells. (A) UMAP highlighting *ZBTB16* (zinc finger and BTB domain-containing protein 16, promyelocytic leukaemia zinc finger, PLZF) gene expression. **(B)** UMAP highlighting *KLRB1* (killer cell lectin-like receptor subfamily B, member 1, CD161). **(C)** Celltypist confidence scores for cells labelled as MAIT cells in data. Red means high confidence, blue means low confidence. **(D)** UMAP of all cells after subclustering cluster 26 using FindSubClusters() into three smaller clusters. Cluster 26_1 (n = 501) was re-labelled as MAIT cells based on the Celltypist confidence and canonical gene expression.
